# From leprosy to ground zero: Imagining futures in a world of elimination

**DOI:** 10.1111/maq.12905

**Published:** 2024-12-17

**Authors:** James Staples

**Affiliations:** ^1^ Department of Social and Political Sciences Brunel University of London London UK

**Keywords:** disease elimination, India, leprosy

## Abstract

Achieving a target of zero—zero disease, zero disability, and zero discrimination—has become the dominant focus of campaigns to control or eliminate diseases, from HIV/AIDS to malaria to leprosy. Given the historical failure of most eradication programs over the last century, such teleological imaginings of disease‐free futures might seem overly utopian. But even if it were possible to eradicate such diseases in their entirety, would this be universally welcomed, even by those most affected by them? In this article, I compare the narratives of national and international bodies concerned with eliminating leprosy, in particular, with the more ambivalent narratives of those affected by the disease in India, the country where the disease is most prevalent. For the latter, the promise of elimination not only seems unrealistic, but represents a potential loss of identity. Imagining disease trajectories in less linear terms, I argue, might also nuance understanding of them.

## INTRODUCTION

The first paper I ever published on my fieldwork in South India began with a quote from Victor—an older leprosy‐affected man I met in a small leprosy colony in Haryana, not far from Delhi. Victor told me: “…us lepers never go whole to the grave. We go piece by piece” (Staples 2003, 295). Although it was a statement made partly in jest—it referenced “leper jokes” about fingers and toes literally dropping off—it also hinted at the fact that for people affected by leprosy (as they came to be known), the trajectory of living with the disease was not unilinear. Leprosy does not kill those who contract it, but left untreated, the disease can lead to various impairments, all of which are experienced against a changing backdrop of sociocultural, political, and economic circumstances. Victor's comment also suggests a certain resistance, both of leprosy itself and of those affected by it, to being eliminated. Leprosy can appear to come and go: people I knew told me about relapses, sometimes years after treatment, which required another round of drug therapy; others talked of “reactions,” memorably described by one of my interlocutors as feeling like “being on fire just under the skin.” “Reactions,” the English shorthand deployed by nearly all my interlocutors in India, referred to patients’ immune responses to the leprosy bacillus, *Mycobacterium leprae*. And although they could occur at any stage of the disease, they were often, as Chen et al. phrase it, “initiated or aggravated by effective chemotherapy due to the active destruction of bacilli during or after treatment” (2022, 4): that is, as a consequence of the very treatment they took to counter the disease and render themselves noninfectious. Treatment for reactions, often with steroids which bring their own side‐effects, might be required for upward of a decade after treatment for leprosy has been completed (White, 2020, 958). Undergoing leprosy‐related treatment—and living and dying as a person affected by leprosy—was not a straightforward progressive trajectory from illness back to good health, in the same way that “cure,” as Venkat (2021) demonstrates in relation to tuberculosis in India, is seldom a final ending.

Thinking about Victor's quote again now, more than two decades on, long after Victor himself passed away, I am struck that his words—counter to the teleological and definitive end‐game narratives that dominate public health discourses—might equally be applied to the disease itself, and to the categories into which it is placed, beyond the bodies and identities of the people affected by it. Leprosy does not just stop existing; neither does it fade away in a linear fashion, cases diminishing on a year‐by‐year basis. One minute—when leprosy was declared by the World Health Organisation (WHO) as globally eliminated as a public health problem in 2000, for example, or when the Indian government celebrated meeting its own elimination targets at the end of 2005—it seems to be on the way out. The next—when case numbers are shown to be rising again—it appears to be back in full force. In 2022, for example—two decades after leprosy was officially eliminated globally—the Leprosy Mission^1^ highlighted that not only was there an 8.67% rise in new cases of leprosy from 2020 to 2021, but that the disability rate on diagnosis had shot up by 15%.

Here, I consider how exploring the case of leprosy in India—the country in which leprosy has been most prevalent—might help to make sense of some of these apparent contradictions. Stories told about leprosy—at least those told by the national and international organizations involved in leprosy management who exert the most overt authority over which narratives are dominant—are oriented as much to an imagined future as they are concerned with the immediate present. These narratives, in common with multiple narratives about AIDS/HIV, malaria, tuberculosis, and other diseases, are stories that end with the disease being vanquished. They are all stories focused on achieving an imagined future of zero new cases: visions that appeal because they offer a “clean slate” and fit well with national ambitions to achieve modernity. Zero targets also appeal to funders because they more clearly define the limits of their expected contribution rather an open‐ended commitment (White, 2020, 958–9).

But how might people affected by leprosy, and those at the front‐end of administering leprosy treatment or implementing policy initiatives, envisage the future in ways that do not fully conform to the dominant aspiration of a world with zero leprosy? And how do these different perspectives on the future interact with one another, and in doing so shape lives in the present and future possibilities? Eradication narratives, I argue here, only fully make sense within particular human‐centered perspectives on the world, within which the good of science, combined with noble human endeavor, can vanquish disease and other evils. As those I worked with understood through experience, such fantasies (as Venkat calls them in relation to narratives of cure [2016]) are problematic because they bracket the negative impacts of modernity and its scientific progress; and disregard the afterlives of diseases like leprosy, which continue to shape people's experience long after they have been officially cured or “eliminated.”

In order to illustrate this argument ethnographically, let me begin by sketching out some of the recent background to leprosy management at global and national scales, and the wider debates around “eradication” and “elimination” within which that management is framed, before honing in more specifically to Anandapuram, the self‐run leprosy colony in South India I have been visiting since 1984, and to the perspectives of the people who live there on a post‐leprosy world: a trope that looms large in the discourse. I consider how they envisage their own futures, as well as that of the disease that has, in large part, defined a collective identity which extends beyond their own colony's borders. This is important because the people affected by leprosy that I worked with had different, more nuanced perspectives on the most desirable outcomes of leprosy management programs than either disability activists or global health programs.

First, however, some brief clarification of terms is required, since, despite their demotic usage as synonyms, “eradication” and “elimination” have very specific—albeit contested—deployments within public health. Here, “eradication” is broadly used in the senses defined by the Dahlem Workshop on the Eradication of Infectious Diseases in 1997, as including either the extinction of a disease pathogen, and/or the “permanent reduction to zero of the worldwide incidence of infection caused by a specific agent as a result of deliberate efforts” (Dowdle, 1998, cited in Patro et al., 2011, 549), with no further interventions required. “Elimination,” on the other hand, was defined as “the reduction to zero of the incidence of a specified disease in a defined geographical area” (Patro et al., 2011, 549), even though continued interventions might be necessary to prevent the disease from reoccurring. The campaign to eliminate guinea worm, for example, even if it achieves its zero goal, will still require work to sustain supplies of safe drinking water to prevent it from reoccurring (White, 2020, 961). Signaling how these terms might also be manipulated, in the case of leprosy, “elimination” was redefined by the WHO as reaching a target of less than one case per 10,000 members of any given population—a seemingly arbitrary figure at which, as the subsequent rise in cases demonstrated, was inadequate to prevent the continued spread of leprosy. Although I use the term “elimination” broadly in line with the WHO's definition—alongside other phrases, such as “end‐game strategies” to encompass other forms of disease control beyond the strictures of either “eradication” or “elimination”—it is also worth noting that many in the leprosy field would contest the very premise that leprosy is a disease susceptible to “elimination” strategies (e.g., Lockwood & Suneetha, 2005; Sanderson, 2008; Smith & Richardus, 2008).

Despite differences of opinion within public health circles, however, disability activists and global health programs have nevertheless tended to take one of two broadly‐defined positions. Either they expressed a commitment to a world *without* disease and disability—utopian projects which funders and bodies such as the WHO, even when they might question the viability of such projects, generally favored—*or*, as was often the case for disability activist organizations, they railed against the potential loss of human difference that eliminating disability categories might entail. For deaf activists in India, for example, state plans to eliminate deafness through cochlear implantation might also be critiqued as attempts to eliminate deaf people themselves (see, e.g., Bauman & Murray, 2014; Friedner, 2022, 17). While such projects have long been contested, however, I suggest here that *both* those who are committed to a world without disability *and* those who lament the potential loss of human difference that this might entail are missing something. Quite apart from the practical question of whether leprosy—or deafness, or malaria, or any of the other conditions subject to end‐game strategies—*can* ever be got rid of, both positions seem to accept that there are currently stable identities—and biochemical/genetic makeups—that, at some point in the future, could at least notionally be made to disappear. From such a perspective, one is, for example, *either* deaf, *or* hearing, blind, or sighted. Leprosy‐disabled or, to use my own interlocutors’ term, “healthy.” It also implies that what it is to be, say, deaf, or a person affected by leprosy, is in itself a materially stable condition.

What my own longitudinal research with leprosy affected people suggests, however, is that these disability categories themselves are in constant flux, and that rather than people's identities simply disappearing or anachronistically remaining—like ghosts of the past—as their conditions are eliminated or controlled, these identities more likely change. This, I shall argue, has important ramifications for how we think about elimination and its implications for people disabled by leprosy and other conditions (notwithstanding the fact that different conditions also have their own trajectories).

Before we get there, I begin with a brief description of my fieldwork site and my research methods, since they provide important context to the understandings of those I worked with that come later. I then go on to offer a brief overview of contemporary leprosy—at least since official global elimination as a public health problem in 2000—before shifting to the perspectives of my own interlocutors and, following that, to how leprosy futures are being configured.

## FIELDWORK CONTEXT

My first visit to the self‐run leprosy community I have called Anandapuram was back in 1984, not as an anthropologist but as a volunteer straight out of high school, whose key roles were to offer English classes to children and to correspond with donors. By the time I returned to Anandapuram to conduct PhD fieldwork in 1999–2000, I had spent over a year there as a volunteer, knew many people who lived there quite well, and communicated with them in a mixture of Telugu (the local language) and English. Since then, in addition to a 16‐month period researching disability in Hyderabad from 2005 to 2006—during which time I regularly took the six‐hour train journey back to the village—I have visited Anandapuram for shorter trips once every couple of years or so. My last visit, in 2022, focused specifically on comparing what people had told me on the cusp of global elimination—when there were genuine concerns about what a world with no leprosy would look like—and how those who remained felt now.

As on my previous visits, I lived in a small house within the village, and spent most of my days hanging out—in tea shops, the clinic, the colony's office, the community hall, on people's verandas and, if invited, inside their compounds and homes. I worked with Das, my long‐time research assistant and a self‐identified “former leprosy patient,” who lived with his own family in the village. Together we conducted a number of informal interviews with members of the diminishing population of people who had themselves been directly affected by leprosy. The village population in 2022 was roughly the same as in 1999—when my own survey (Staples, 2003, 297) counted 907 residents—but in those days around half of them had previously been diagnosed with leprosy, and around 300 had leprosy related disabilities. Now, fewer than 200 people affected by leprosy remain. Of those, fewer than 100 were leprosy disabled, and their bodily differences were significantly less obvious than those I encountered more than two decades earlier. While many “patients” in 2000 were identifiable by their collapsed noses, and clawed or missing fingers and toes, in 2022 most could pass as what they referred to as “healthy” people—a broad category covering everyone *without* leprosy.

We also met contacts in other leprosy organizations, including a visiting cobbler, who was employed by an international NGO to visit leprosy colonies and hospitals to make special footwear for those with leprosy damaged feet; a leprosy physio‐technician; a visiting medical doctor; and leprosy fieldworkers whose task was to identify cases and refer them on for treatment.

While there is perhaps no such thing as a typical leprosy colony, Anandapuram differs from NGO‐or government established colonies and leprosaria in that it was self‐established and remains self‐governing. Its first settlers arrived on what was then a patch of railway wasteland in the late 1950s, when advances in leprosy drug treatment allowed long‐term patients to be discharged from a local residential mission hospital. Unable to return to their home communities for multiple reasons—including religious conversion to Christianity, marriage to fellow patients outside their castes, and fears of being stigmatized because of their disease—Anandapuram's first generation relied mostly on begging to make an independent living (Staples, 2007).

The early settlers also registered themselves as an Association under the Societies’ Act, with Elders elected from the membership. Being a man affected by leprosy was a condition of membership, ruling out most subsequent generations (and all women) from officially joining the Association.

From the late 1970s until the mid‐1990s, there was also a foreign presence in the village, with overseas volunteers working in conjunction with the Elders to set up and run a variety of social welfare and development programs, including income generation schemes, a clinic, a creche, and primary school.

As on my previous visits, I also kept daily field notes. Ethical permission for the field work was granted by Brunel's research ethics committee,^2^ and I followed—as on previous visits—the Association of Social Anthropologists (ASA's) ethical guidelines in conducting fieldwork. My interlocutors were made aware of my current project, and consent was negotiated as an ongoing process. All names used are pseudonyms, to protect individual anonymity.

Although this article draws in particular on my most recent trip to Anandapuram, it would not have been possible to write it without the longitudinal perspectives gained from visiting the same community over a nearly 40‐year‐period. In 1984, almost everyone in the village lived in mud and thatched huts, at least one member of every family had been directly affected by leprosy, and a large number of residents relied on begging for income. These days, there are far fewer people who have ever been diagnosed with or treated for leprosy, even though they are all, in certain ways, affected by leprosy. Those who remain are less obviously physically changed by their disease than those I knew in the 1980s and early 1990s. They also, as I show in the following, have different experiences of living with the disease. Before getting to that, however, I want first to zoom out and offer a sketch of the wider national and international perspectives on leprosy within which Anandapuram and its residents were embedded.

## THE LEPROSY SCENE

The Global Partnership for Zero Leprosy (GPZL), launched in 2018, brings together a coalition of organizations and individuals from across the world who are committed to the teleological endeavor of “ending leprosy,” and currently promotes what it describes as a “triple‐zero vision of no disease, no disability, and no discrimination or stigma” (https://zeroleprosy.org/). This “triple zero” vision aligns with that established for several other targeted diseases, including, for example, the one set out in the United Nation's vision for AIDS/HIV, which is also “to achieve three zeros: zero new HIV infections, zero AIDS‐related deaths and zero discrimination” (Global AIDS Update 2022, 42).

However, as noted above, elimination of leprosy as a public health problem globally had already been officially achieved in 2000, during the first period of my prolonged fieldwork. Elimination, in these terms, was not achieved in India at a national level until the end of 2005, when its national prevalence rate dipped to 0.96, and in most other countries by the end of the decade. “India achieves leprosy eradication target,” read a headline in *The Hindu* newspaper on January 31, 2006, below which—under the caption “Fighting the scourge”—India's then‐president, Abdul Kalam, was pictured inaugurating the Anti‐Leprosy Fortnight in New Delhi. Buried in the fourth paragraph of that same story, however, was the fact that six states—between them making up more than 40% of the population—still had prevalence rates of between 1 and 3 per 10,000. In the same way that global averages detracted attention from troubling national leprosy statistics, so did nationwide figures miss the point that plenty of new cases were still being identified in parts of the country.

Hopes of governments, INGOs, and NGOs of consigning leprosy to the history books were short lived. In 2009, of the 244,769 new cases reported across the world, 133,717 of them still came from India (Patro et al., 2011). Newspapers and other media that had celebrated India's success in bringing leprosy under control in 2006 now expressed horror that cases were still emerging at an apparently high rate and rising. By 2016, India was reporting an annual 135,485 new leprosy cases, up from a low of 127,326 in 2015 (Rao & Suneetha, 2018, 4). The latest figures were almost unchanged from 2007 (which saw 137,685 cases reported), and seemed to add weight to the argument that elimination was not in itself enough to end leprosy as a public health concern (Rao & Suneetha, 2018, 4). There have also been serious concerns over the accuracy of case detection data, with fears that new cases been significantly under‐reported since leprosy as a global health problem was officially eliminated (Smith et al., 2015; White, 2020, 957). The dramatic fall in new case detection rates between 2001 and 2005, of more than 60%, run counter to disease modelling that predicted, under good circumstances, a decline of just 4.4 per cent per year (Smith et al., 2015, 2). The WHO's new strategy—running from 2021 to 2030—goes beyond the previous elimination targets, aiming not only for no new cases of leprosy, but also zero disability and zero discrimination.

For most of the people I worked with, however, leprosy—and people affected by leprosy—were seen as here to stay, at least for the foreseeable future, and attempts to cut back funding or scale back had negative effects on leprosy affected people in the present. “It's just there in the air,” as one woman, a cured leprosy patient, told me, when I was back in the Anandapuram in summer 2022. “You can't get rid of the air, so I don't think you can get rid of leprosy.” And Mohan Rao, a paramedic who himself had leprosy impairments, reflected the views of many with his comment:

It's really not possible to get to zero leprosy, whatever they say. Resistance to the drugs will increase, and then numbers will rise again. I've seen the children of people affected by leprosy testing positive after 15, 16 years of testing negative. Patches come, then reactions. We can treat, and if we treat quickly it will be okay, but also people will be neglectful. They might not come for treatment early, or they might not take the medicines properly. So, can we achieve Zero Leprosy? No chance.

There are other challenges, too—many of which are acknowledged by the WHO and members of GPZL. As another leprosy paramedic whose parents were both affected by leprosy complained, every time the number of leprosy cases drops, or at least is made to appear as though it is dropping, with it drops the will to act. Pharmaceutical companies, faced with a diminishing market, lose their focus on finding a cure beyond the tried and tested; investment in a vaccine declines, and funds to NGOs, hospitals and others involved in the leprosy field plummet. The employment of Shankar, an old friend who works for an international leprosy NGO in an Indian city, has been precarious since the official elimination of leprosy as a public health issue. After secure employment up until 2000, now he waits nervously every year to see if his contract will be renewed. Funding for his field activities—which involved going house‐to‐house to screen for leprosy cases—has sharply declined. In Anandapuram, Mohan Rao, too, noted that “cases are rarer, but they are still coming. And I know so many patients who have relapsed, sometimes because of neglect, but sometimes it just happens: their immunity is down, and they get reactions, sometimes after 15 or 20 years. And the government hospitals, they won't always take proper care.”

“Sometimes,” interjected Das, my research assistant, “they don't even admit patients who they think have leprosy… When there were separate leprosy hospitals the people who worked there were used to it, knew what to expect. But doctors and nurses in government hospitals, they often know less than we do about leprosy.” While bringing leprosy under the remit of general hospitals has made treatment available at more locations, the parallel decline of specialized centers has also meant that people affected by leprosy are less likely to receive specialist follow‐up care to prevent disability. At the same time, expertise on leprosy, as the WHO also acknowledges, is also in decline. What trainee doctor, after all, choses as their specialism a condition that is hailed as being on the way out, particularly when being a leprosy doctor carries its own stigma (see, e.g., Staples, 2007, 94)?

Biomedicine, the pharmaceuticalization of healthcare, and technological solutions to disease management once appeared to offer opportunities to rid the world of diseases, especially those, like leprosy, thought to be consigned to a premodern era. As Elisha Renne's work on the politics of polio in Nigeria demonstrates, however, a focus on the disease rather than the sociocultural contexts in which it spreads (2010, 13), alongside what Farmer dubbed “the pathogenic role of inequity” (2004, 20) not only renders targeted diseases resistant to elimination but allows newer ones, like AIDS/HIV and COVID‐19, to emerge and spread more quickly than their predecessors.

This, however, is not simply a frustrating story of those with resources, whether individuals, foundations, national governments, or international bodies, not moving fast enough to unleash the full potential of science to make a meaningful difference. Rather, as Leach and Dry (2010) have pointed out, the very conditions of modernity that allowed scientists, global health organizations and philanthropists to imagine, however tentatively, a future free of disease, are also inextricably implicated in how diseases, new and old, have continued to emerge, spread and mutate. The solutions identified to global health problems are already a part of the very problems they set out to solve. Global transport, communication, science, and technology have also “created a unique contemporary vulnerability to novel threats that have the potential to create unprecedented systemic failures at a global level” (Leach & Dry, 2010, 246–7). In the age of the Anthropocene, the COVID‐19 pandemic and the ongoing climate crisis might both be seen as instances of the *negative* impact of modernity; an impact which in turn undermines the narrative of unilinear progress on which the dream of elimination draws.

Nevertheless, the idea of eliminating disease, despite the dubious potential of achieving it, is popularly accepted as something to strive for. After all, a world without disease would, in the words of the International Task Force for Disease Eradication (ITFDE), “dramatically and permanently improv[e] the quality of life for many millions of the world's poorest people.”^3^ As the historian Nancy Leys Stepan points out, however, many in public health *do* question whether the pursuit of eradicating diseases is the most effective way of alleviating suffering. In her book *Eradication*, she asks:

What are the costs as well as the benefits of eradication? Is the concept of the complete extinction of a biological agent or a disease in some kind of tension or even in contradiction with modern environmentalism and ecology? Has eradication become “eradicationism,” or a utopian dream, rather than a practical solution to ill health in human populations? (2011, 9).

End‐game programs—whether for diseases, disabilities, or invasive animal and plant species—might also have unforeseen consequences, as Paolo Bocci's account of a project to save tortoises on the Galapagos islands from extinction captures very eloquently. The program he describes required action to eliminate goats from the islands, something which required the killing of hundreds of thousands of them, and left local inhabitants feeling as though they were in the center of a war zone. This had multiple implications: including the loss of a human food source, the growth of new invasive plant species rather than the envisaged return of native plants, and the continued presence of many goats (Bocci, 2017). Similarly, many of the US Department of Agriculture's (USDA) pest eradication campaigns in the latter half of the 20th century—aimed variously at Dutch elm disease, the gypsy moth, and white fringed beetles—not only failed to eradicate their targets, but have had severely negative consequences for birds, livestock, and pets (Rollason, 2023). Could the eradication of the vectors of diseases—or the bacteria that cause them—similarly have ecological implications beyond those envisaged? Eradication of a disease to protect one species, after all, often demands the extinction, or at least control, of another: goats for turtles, as in Bocci's example, or mosquitos or *Mycobacterium leprae* for people with malaria or leprosy, respectively.

## ELIMINATION OF IDENTITIES?

Against this background—which suggests that the imminent demise of leprosy is unlikely—the question that the anthropologist and disability studies scholar Michele Friedner posed to me during a discussion about elimination of disability and disease, and which provoked this article, seems rather hypothetical. She asked me: “Would those you work with like to see a world in which people like them do not exist?” Her own interest in the question came from her work with deaf people in India, where various states are working to be “deafness free” by 2025. The Indian government, alongside NGOs and charities, has been funding expensive cochlear implants, with a focus on curing deafness and making deaf children almost, near‐to, or perfectly normal. For many deaf people, however, who value “deaf culture” and who promote sign language as a mode of communication on par with spoken languages, eliminating deafness is problematic. The significant lifelong cost of maintaining cochlear implants, even when the state foots the bill for getting them implanted in the first place, posed a different and additional challenge, sometimes leaving the children who had them worse off than had they, as otherwise would have been the case, learned sign language (Friedner, 2022).

When I was conducting my own fieldwork, on the cusp of official elimination in 1999–2000, this loss of identity was certainly a concern for my interlocutors. Many I spoke with that year expressed their anxieties about what would happen to them once leprosy was declared resolved as a global health problem. Would they, for example, still be accepted as worthy recipients of aid when they went begging—which, at the time, was the largest source of income for most families—to major cities? Would charities, like the one I was involved with in the United Kingdom, still be able to raise funds to support social welfare and development programs in the community if people started thinking of leprosy as having been resolved? And would the empathy between leprosy affected people and their families, which living in a leprosy colony enabled, be diminished when there were fewer people left who shared comparable experiences? I have written elsewhere, for example, about the children of parents disabled by leprosy knowing instinctively—in ways that I did not—when and how to assist those without functioning fingers to eat food, smoke a cigarette, or plait their hair (Staples, 2003, 309). Once the chains through which the expert knowledge of patients and those around them are broken, it becomes difficult for them to be re‐established. Carolyn Sargent and Grace Bascope offer a neat example of this in their account of childbirth among poor women in hospitals in Kingston, Jamaica, in the 1990s (1996, 226–7). Century‐long efforts to eradicate lay‐midwifery in order to medicalize birth in Jamaica had meant that midwifery was no longer widely available. When low‐income pregnant women turned to the public hospitals they had been encouraged to attend, however, a lack of funding and shortage of qualified staff—a situation exacerbated by World Bank supported structural adjustment policies—meant that their deliveries were often unaccompanied and in poor conditions.

Anxieties about the loss of empathy, local knowledge, and resources, were not limited to the cured, but still in many cases physically disabled, people affected by leprosy that I spent time with. Senior officers I attended meetings with from international leprosy organizations were worried about the future of their programs and their jobs. A Government leprosy worker, whose job it was to distribute leprosy drugs to those who needed them, discussed with me his colleagues’ fears that, with leprosy being integrated into general medical care, their own posts would be downgraded to those of multipurpose health workers (Staples, 2007, 94). Many others I met in leprosy NGOs were openly discussing the kinds of options that would be open to them post‐leprosy, hopes pinned on the causes of AIDS/HIV or cancer, to which their skills might be transferable. The capacity of modernity to usher in newer diseases to replace those brought under control at least kept the healthcare NGOs in business, even if they needed to remain alert and nimble‐footed if they were to compete successfully with other NGOs for limited resources.

Some of the fears that were being expressed back then certainly came to pass, suggesting that my interlocutors were quite skilled at predicting their own futures. Funding did decline dramatically, with Anandapuram's clinic no longer able to provide the level of care to all villagers that it once had. The closure of local leprosy hospitals was lamented by interlocutors who said they now had to face not only the prejudice of medical staff in general government hospitals but also a lack of knowledge about their disease. Like deaf people in India in the contemporary moment, people affected by leprosy in 2000 feared a loss of identity. Fast forward to the present, however, and the people I still work with in Anandapuram—where fewer than 200 of the 1000 population now identify as people affected by leprosy—imagine the future in rather different ways. Their experience, while particular to their own circumstances, might provide food for thought for others with disease or disability related identities earmarked for elimination.

## ENVISAGING LEPROSY FUTURES IN THE CONTEMPORARY MOMENT

Answering the question of whether my interlocutors would like to see a world in which people like them did not exist proved a more complex task than I might have originally thought, for a number of related reasons. For one thing, what it meant to have leprosy has *literally* changed over the past century. Leprosy was once incurable—eventually burning out, usually after the bodily changes it wrought had identified the person irrevocably as having the disease—in the same way that HIV/AIDS in the 1980s had once been a death sentence before joining a longer list of conditions that could be managed. Medical explanations of cause and cure had likewise come to dislodge, if not entirely replace, the many and variable earlier ideas about cause recorded in relation to leprosy (see, e.g., de Bruin, 1996; Staples, 2007; Wise, 1845). Effective treatment, which also rendered patients noncontagious, had also materially affected the experience of having leprosy. The older generation of people I met in Anandapuram in the 1980s were (although I had ceased to notice it until I looked back at old photographs) visually striking as leprosy patients: collapsed noses, missing eye brows, and fingers and toes clawed, disfigured, or amputated because of injury, as well as leprosy ulcers, were all extremely common. All had received treatment and were noncontagious, but that treatment had often come too late to prevent impairments. Interviewing people in the village in 2022, by contrast, the signs were generally more subtle: a patch of pale skin or a single bent toe or finger, for example, which, to the uninitiated, might well be missed. And while this group retained a sense of kinship with the community's founders—people with whom they shared a diagnosis—that aspect of their identity was less significant than it once would have been. Disabilities and impairments that *did* exist were often unrelated to leprosy. When I asked people, as part of a conversation about their experience of leprosy, whether they still considered themselves “patients,” they would respond that they were, but—after a pause—because of diabetes, high blood pressure, or gastric problems, *not*, in most cases, because of leprosy. What it means materially to be disabled by leprosy has also shifted in the last few decades.

In addition, the “people like them” referenced in the original question is also a tough one to pin down. People affected by leprosy also identify as parents (or children and siblings), as beggars (or rickshaw drivers, engineers and lawyers), as elderly or young, as poor or wealthy, as men or women (or, in a couple of cases, as hijra or—again indexing a modern, global category that has slipped into common parlance—transgender), and as sugar, stroke, and BP patients. One of things that caused me to panic in the early stages of my PhD fieldwork, which was focused on capturing the lived experience of leprosy, was that people living in a leprosy colony hardly ever talk about leprosy at all. And these other, intersecting identities are also unstable, multiple, and liable to change across spaces and at different times. Indeed, my second book, *Leprosy and a Life in South India*, attempts to tackle a problem inherent in writing about disability, disease and difference: placing whatever condition it might be at the center of our analyses might often stand in tension with how our interlocutors might see themselves in relation to the world. For Das, the protagonist of that book (a biography), leprosy had played a major role in how his life had panned out, but it was only one of several competing factors that shaped what happened to him from the late 1960s onward (Staples, 2014, xvii). To quote myself: “Leprosy and people's reactions to it, I discovered, shoulder the blame for a whole range of social ills and provide cover for still more uncomfortable realities” (Staples, 2014, xvii). Elimination of leprosy would certainly destabilize how people identified themselves, but it would be only one of a number of moving parts through which personhood was dynamically constituted—particularly in a community that was organically transitioning from being a leprosy colony to being a village, albeit one shaped by the memories of living with a disease.

In addition, the fears that been evoked by the imminent elimination of leprosy at the start of the 21st century have somewhat subsided. Leprosy, most of those I spoke to seemed to think, was here to stay, certainly beyond their own envisaged lifetimes. Their acceptance that leprosy was unlikely to be eliminated was not a pessimistic view, however. As they put it, as long as leprosy remains, awareness, and thus resources, are less likely to diminish. They knew, from the experience of 2000, that declaration of success in meeting targets leads to a decrease in leprosy funding and a decrease in the visibility of their continued plight. Dr. Fred Lowe Soper (1893–1977), the arch‐eradicationist through whom Stepan tells the history of eradication as an idea, recognized that:

“…to accept eradication as a goal in public health implied a fundamental shift in psychology or in attitude toward disease, especially at the point when a disease targeted for eradication has almost disappeared; this is when most people tend to lose interest in continuing on to complete the job of eradication, and instead shift their interest in continuing on to other pressing public health tasks” (Stepan, 2011, 16).

So it was with leprosy, despite the endeavors of international public health bodies, from the “Final Push Strategy” that directly followed elimination to the current “Global Partnership for Zero Leprosy”: interest and funding waned, and case numbers again rose. As such, there will be no single moment of cut‐off, no final ending to the story. As with pandemics, like COVID‐19, there will likely be a gradual drift from front‐of‐stage toward the wings, with the occasional return to the center of the stage to take a curtain call.

My interlocutors are not, however, mourning the diminishing numbers of people clearly identifiable because of their distinctive impairments as having leprosy; much as they valued the shared experience of having people around them who understood the sociophysical constraints that leprosy, and reactions to it, gave rise to. Many found it difficult to articulate it specifically, but they expressed a sense of nostalgia for a time when neighbors—united in their shared experience of leprosy if not always much else—had more readily helped one another with mundane tasks, often without the need for asking. Such nostalgia for these past aspects of a shared leprosy identity, however, was inseparably bound up with other memories from the same era, when chickens tasted like chickens and men gathered to listen to the radio in the central teashop rather than watch it on TV in the increasing privacy of their own houses and walled‐in compounds. That is to say, those memories were part and parcel of a wider set of selective positive memories that romanticized the past.

It would, most conceded if pushed, be better *not* to have leprosy affecting new people. What they *would* miss—and their healthy children also feared this—would be the loss of other, related identities. As people affected by leprosy, they identified as a people who had a right to ask for support; as worthy recipients of aid; as targets of support from Christian (for which also read overseas/international) organizations. My interlocutors were no keener now than they had been 40 years earlier simply to be reinserted back into society in the social positions they had occupied pre‐leprosy, since, in most cases, these were already negative ones. What external NGOs sometimes perceived of as the indignities of being a recipient of welfare in a leprosy colony by virtue of one's disease status was often recognized by those recipients as preferable to the indignities of a life as a low‐status, daily‐wage laborer.

Even these associated identities, however, are in flux. Take, for example, the generation of healthy younger people that I observed struggling during my 1999–2000 fieldwork. Many of them, thanks to links to leprosy‐related NGOs, had been educated beyond the expectations of families mostly constituted of farm laborers. But without the social capital to trade that education for suitable employment, and without the knowledge or bodies to beg successfully—as many of their parents did—they had struggled to find a place in society. Many of those who were not highly educated at that time (and a few who were) became rickshaw pullers instead of taking up begging, like their parents, but remained poor: “We've become a colony of rickshaw wallahs!” as I recall one woman joking to me back then. And while some tentatively described this as progress—it was seen as more socially acceptable than begging, albeit less lucrative—a spike in suicide and attempted suicide rates among young people in the community suggested a more complex picture (see, e.g., Staples, 2007, 2014).

Now, fast forward to the 2020s. That generation has been replaced by another: the children, grandchildren and sometimes great‐grandchildren of people who had been diagnosed with leprosy. This generation is also educated (often more highly), but, thanks to a few successful pioneers, some now *do* have the capital, albeit limited, to get the jobs that often eluded their predecessors. The wider context, marked by an increasing number of jobs available in call centers and back offices for international organizations, has also helped, as has the gradual decline—largely through the dying out of earlier generations—in numbers reliant on begging. As the area in which the leprosy colony is located becomes increasingly heavily populated, the once firm boundary between it and the outside world has also been eroded, with outsiders *without* leprosy buying up land, and building homes, in the spaces between the village and the local town. This new generation, then—more distanced from the direct experience of leprosy—has potentially benefitted from opportunities offered by NGO and state‐sponsored programs that would not have been so readily available outside the context of a leprosy colony, but, crucially, they now also have routes to utilize those benefits. Instead of lobbying the NGO's office for opportunities for their children, or trying to find the money for the bribes that used to be required for even the most low‐level government jobs, there is perhaps a recognition that it makes sense to communicate with those who have already found private jobs with major firms in Hyderabad and elsewhere. In the 1980s, a government job was the ultimate fantasy for most. Now, liberalization of the economy has shifted aspirations to focus on getting jobs in large, private companies. Competition remains fierce, but—armed with their qualifications—some of these opportunities are at least achievable for some young people in Anandapuram.

## CONCLUSION

Trajectories of diseases like leprosy are not, as my interlocutors recognized, as unilinear as the dominant narratives of global health initiatives sometimes present them as being. As such, strategies to eliminate such diseases in accordance with rigid timelines are unlikely to succeed—a prediction supported by the chequered history of disease control programs over the last century. That is not necessarily to condemn such programs. In doing their job of focusing attention on a problem and attracting both resources and research, leprosy elimination programs have done a great deal to reduce the overall numbers of people who contract leprosy globally every year, and they have played a not insignificant role in changing the physical reality of being a person affected by leprosy in the contemporary moment. Those I worked with in 2022 were far less likely to be obviously physically disabled by leprosy than their counterparts in 1999–2000, and they consequently faced less social stigma and fewer material restrictions.

At the same time, however, on‐the‐ground local experiences of living with leprosy and other conditions subject to end‐game strategies also question the desirability of such futures. Elimination of a disease, as I have shown in this article, also entails elimination of categories through which forms of “biological citizenship” (Petryna, 2005) have been made possible. Elimination, as we have also seen, also often entails the privileging of one life form over another. Even if there were to be a consensus that human life is worth more than that of a *bacillus* (one to which, as a human being, I subscribe), and that such a *bacillus*, which has been successfully evolving for millions of years, *could* be eradicated, things are still not straightforward. The elimination of other species, as we can see in relation to Paolo Bocci's (2017) account of attempts to eradicate goats in the Galapagos islands in order to save tortoises, often bring negative unforeseen consequences, such as the proliferation of other, perhaps even less welcome, species. The fact that *Mycobacterium leprae* and its ancestors have been present on earth for more than 20 million years (Singh et al., 2015)—significantly longer than modern human beings—might also give us pause for thought, not only about human exceptionalism and the right or otherwise of particular species to exist, but also about the practicalities of eliminating other, rapidly evolving life forms.

What I also hope to have demonstrated is that disability—or any particular named condition—is never experienced outside of the contexts in which it is relationally defined. In that sense, there is never an objective or uniform way in which leprosy is experienced as an identity category. Elimination campaigns do not adequately reflect this nuance. Changes in name, changes in the condition itself and its responses to particular treatments and other interventions, and the ebbs and flows of other identities that intersect with this identity all have implications for the lived experience of leprosy (something which elimination campaigns tend not to be concerned with). If I were to interrogate my respondents’ comments about the future to address the question—do you want a world without people like you in it?—I think the answer would be a cautious yes: just not yet, nor too quickly. And the implications of that for policy, I would suggest, are that we might do better to focus on managing the trajectories of diseases over time rather than, as has more often been the case, fixating on the goal of zero.

## ENDNOTES

1
https://www.leprosymission.org.uk/latest‐news/low‐numbers‐of‐people‐treated‐for‐leprosy‐since‐2020‐are‐extremely‐concerning/#:~:text=New%20World%20Health%20Organisation%20figures%20show%20that%20the,hoped%20the%202021%20figure%20would%20be%20significantly%20higher.

2 Brunel Research Ethics online Application Number: 36679‐MHR‐Jul/2022‐ 40581‐3

3
https://www.cartercenter.org/health/itfde/index.html
